# Experimental and analytical datasets for pressure drop and flow distribution in a Z-type flat-plate solar absorber

**DOI:** 10.1016/j.dib.2022.108162

**Published:** 2022-04-10

**Authors:** Alireza Shantia, Wolfgang Streicher

**Affiliations:** Unit of Energy Efficient Buildings, Department of Structural Engineering and Material Sciences, University of Innsbruck, Innsbruck, Austria

**Keywords:** Flat-plate solar collector, Z-type solar absorber, High-resolution pressure measurements, Flow distribution

## Abstract

This data article provides two dataset types for total pressure drop and parallel-flow distribution in a Z-type flat-plate solar thermal absorber with water as the working fluid. The first dataset consists of high-resolution pressure drop measurements at different temperatures under laminar and turbulent flow conditions obtained experimentally using a state-of-the-art hydraulic test rig. The second dataset comprises analytical data on flow distribution in the absorber. Conducting high-resolution pressure measurements, essential for evaluating thermo-hydraulic models, is a sensitive and time-demanding process requiring a relatively elaborated test rig to accurately measure pressure drop at different temperatures and flow rates in the presence of thermal equilibrium. In this context, engineers and researchers can use these datasets to compare and verify developed numerical models for thermo-hydraulic evaluation of pressure drop and flow distribution in flat-plate solar collectors under both laminar and turbulent flow regimes. The article also comprises analytical data for flow distribution in the absorber for several header configurations presented by dimensionless-flow-rate and non-uniformity. This data article is related to the research article (Shantia et al., 2022 ). The datasets are accessible in the supplementary files accompanied by the online version of this article and in the Mendeley Data repository.

## Specifications Table

**Table:** 

Subject	Renewable Energy; Sustainability and the Environment
Specific subject area	Thermo-hydraulic analysis in flat-plate solar collectors
Type of data	Table, figure, schematic, MS-Excel Spreadsheet
How data were acquired	The pressure data were obtained from the developed test rig for high-resolution pressure drop measurements. The flow distribution data were acquired using the numerical model presented in [1] for thermo-hydraulic analysis.
Data format	Raw, analyzed
Description of data collection	The data were collected for water as the working fluid under thermal equilibrium using a differential pressure sensor Deltabar M PMD55 from Endress+Hauser and a KROHNE OPTIFUX 2000 electromagnetic flow meter. The data range is in the interval 0.05-0.98 m^3^ hr^−1^ for flow rate, 0.078-8.197 kPa for total pressure drop, and 20-80°C for inlet temperature.
Data source location	The measurements data were obtained from the experimental setup in the hydraulic lab of the Unit of Energy Efficient Buildings Institution: Department of Structural Engineering and Material Sciences, University of Innsbruck City: Innsbruck Country: Austria
Data accessibility	The data are available within the online version of this article and also in a repository at https://data.mendeley.com/datasets/wvhpf2hp8z/1
Related research article	A. Shantia, W. Streicher, C. Bales, Effect of tapered headers on pressure drop and flow distribution in a Z-type polymeric solar absorber, Sol. Energy. 232 (2022) 283–297. https://doi.org/10.1016/j.solener.2021.11.048

## Value of the Data

• The experimental data on total pressure drop is essential to verify mathematical models used for thermo-hydraulic analysis in flat-plate solar collectors.
• Using these high-resolution datasets with a wide range of flow rates and temperatures provides the possibility to examine all flow regimes from fully laminar to fully turbulent.
• Researchers can use the datasets to benchmark pressure drop and parallel-flow distribution in flat-plate solar collectors resulting from fluid dynamics and geometrical characteristics.
• The data can be used to gain insights into pressure drop and flow distribution characteristics in Z-type flat-plate solar collectors to improve flow distribution features for thermal performance betterment.

## 1 Data Description

**Fig. 1:**
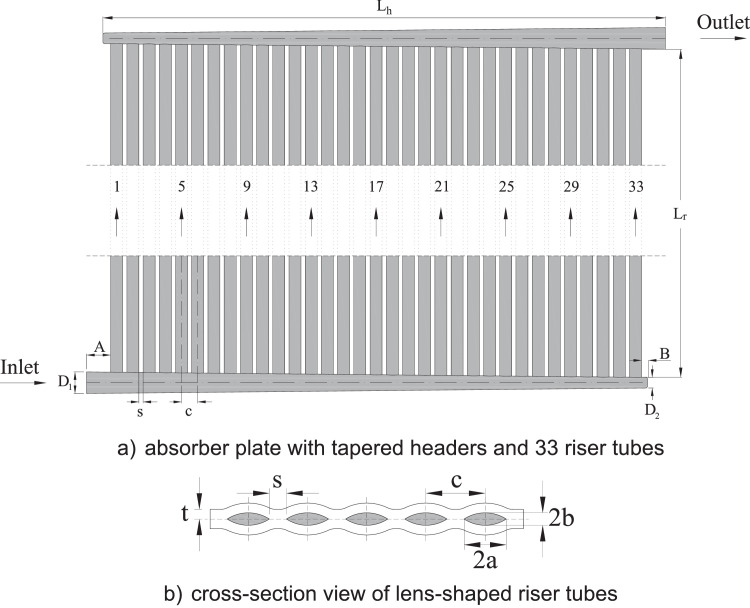
Schematic view of the Z-type absorber (reproduced from Shantia et al. [1]).

This paper presents benchmark datasets for pressure drop and flow distribution in a flat-plate solar thermal absorber with Z-configuration in the headers, as shown in Fig. 1(a). The absorber consists of 33 parallel strings with identical lens-shaped cross-sections illustrated in Fig. 1(b). The diameter linearly reduces from 22 mm (*D_1_*) to 8 mm (*D_2_*) in the inlet header while decreasing in the same manner in the outlet header, giving a convergence angle of 1.73° in both headers. Detailed geometric attributes of the absorber are reported in Table 1.

**Table 1:** Geometric attributes of the absorber shown in Fig. 1 (reproduced from Shantia et al. [1]).

Total area (length x width)	0.422 m^2^
Polymer sheet thickness (*t*)	2 mm
Header length (*L_h_*)	463 mm
Header inner diameter max. (*D_1_*)	22 mm
Header inner diameter min. (*D_2_*)	8 mm
Header convergence angle (θ)	1.73°
Header Inlet length (*A*)	20 mm
Header dead length (*B*)	5 mm
Number of risers (*nr*)	33
Riser tube length (*L_r_*)	927 mm
Lens-shaped inner length (*2a*)	10 mm
Lens-shaped inner width (*2b*)	3 mm
Riser hydraulic diameter (*D_r,hyd_*)	3.84 mm
Riser inner cross-sectional area (*A_r_*)	20.36 mm^2^
Distance between tubes (*c*)	13.5 mm
Riser intermediate spacing (*s*)	3.5 mm

This article is accompanied by spreadsheet files with the same name as the figure number in the originating paper [1]. For example, the data for “Fig. x” in [1] is listed in the file “Fig. x_Data.” The datasets are provided as supplementary materials in MS-Excel format (∗.xlxs) accessible in the online version of this paper and Mendeley Data repository. In addition, a detailed description of the variables together with units and data extraction procedure is reported in Table 2.

**Table 2:** Description of dataset variables.

Variable	Description	Unit	Source/Procedure
*T_in_*	Absorber inlet temperature	°C	Measured by RTD sensor (Pt100)
*T_out_*	Absorber outlet temperature	°C	Measured by RTD sensor (Pt100)
*T_mean_*	Absorber mean temperature used for thremo-hydraulic evaluations	°C	= (T_in_+T_out_)/2
*V*	Volume flow rate	m^3^ hr^−1^	Measured by KROHNE OPTIFUX 2000
*Δp*	Absorber total pressure drop	kPa	Measure by Deltabar M PMD55 differential sensor
*α*	Dimensionless flow rate, α	–	Eq. (21) in Shantia et al. [1]
ϕ	Non-uniformity	–	Eq. (22) in Shantia et al. [1]

## 2 Experimental Design, Materials and Methods

**Fig. 2:**
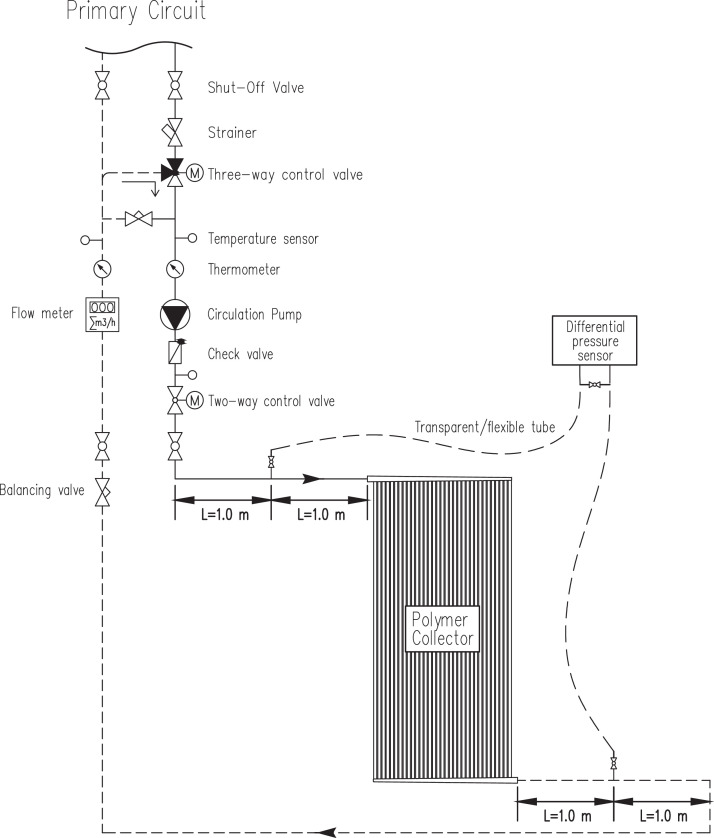
Experimental setup for high-resolution pressure drop measurements (reproduced from Shantia et al. [1]).

The experiment setup for total pressure drop measurements is shown in Fig. 2. The experiment boundary conditions are listed in Table 3. The hydraulic test bench is equipped with a two-way and a three-way control valve, a variable speed pump, a balancing valve, a volume flow meter, a differential pressure sensor, and three temperature sensors. This relatively elaborated test rig allows high-resolution pressure measurements, which is essential for assessing all flow regimes from fully laminar to fully turbulent. The measurements range is in the interval 0.05–0.98 m^3^ hr^−1^ for flow rate, 0.078–8.197 kPa for total pressure drop, and 20–80 °C for inlet temperature.

**Table 3:** Experiment boundary conditions for total pressure drop measurements.

Measurement ranges	
Medium	Water
Absorber tilt angle	0° (horizontal)
Inlet temperature range	20 °C, 40 °C, 60 °C, 80 °C
Flow rate range	0.05 m^3^ hr^−1^ to 0.98 m^3^ hr^−1^
Pressure drop range	78 Pa to 8.917 kPa

Differential pressure sensor characteristics	
Manufacturer	Endress+Hauser
Model	Deltabar M PMD55
Nominal accuracy	±5 Pa

Volume flowmeter characteristics	
Manufacturer	KROHNE
Model	OPTIFUX 2000
Type	Electromagnetic
Nominal accuracy at 0.05 m^3^ hr^−1^	±3%
Nominal accuracy at 0.98 m^3^ hr^−1^	±1%
Nominal accuracy of RTD temperature sensors	Pt100: ±0.05 °C

The differential pressure across the absorber is measured indoors with no solar irradiance to avoid significant temperature variations. During the experiment, the absorber was horizontally laid on the ground, and temperature gradients across the absorber were kept relatively small (<25 °C) in order to eliminate the impact of buoyancy forces on the measurements even at low flow rates under laminar regimes. The inlet temperature to the absorber is regulated by a three-way mixing valve and an RTD (Resistance Temperature Detector) sensor in close vicinity. The flow rate is precisely adjusted by manually setting the variable speed pump alongside the two-way valve upstream and the balancing valve downstream. Note that the fluid flow is not entirely isothermal. In fact, the temperature drops up to 25 °C can still occur in the worst case (i.e., highest temperature/lowest flow rate) in the absorber outlet due to heat losses from the absorber. The instantaneous values of the monitored data, including overall flow rate, differential pressure, and temperatures, were recorded once per second in the presence of thermal equilibrium under which the outlet temperature remained constant. The mean values of the measured temperatures at the absorber inlet/outlet were then used to determine the thermophysical properties of water in all hydraulic components to produce the analytical data.

The total mass flow rates were calculated based on the downstream temperature and measured volume flow rate nearby via KROHNE OPTIFUX 2000 electromagnetic flow meter. The accuracy of the flowmeter is within ±1% for the upper range (0.98 m^3^ hr^−1^) and ±3% for the lower range (0.05 m^3^ hr^−1^). In addition, the total pressure drop is measured with a differential pressure sensor Deltabar M PMD55 from Endress+Hauser with a nominal accuracy of ±5 Pa. A manual air vent integrated into the pressure sensor together with transparent plastic tubes between the collector inlet/outlet and the pressure sensor (see Fig. 2) ensures no air in the test rig within measurements. In order to establish a fully developed flow at pressure measuring points, the transparent measurement tubes are connected in the middle of a straight pipe (two meters long) at both the upstream and downstream end of the absorber. Finally, to retain the accuracy of the flow meter and differential sensor within the values mentioned above, the measuring range in both instruments is rescaled for each temperature set in three steps: (1) *V* < 0.12 m^3^ hr^−1^, *Δp* < 0.50 kPa; (2) *V* < 0.40 m^3^ hr^−1^, *Δp* < 2.50 kPa; (3) *V* < 1.0 m^3^ hr^−1^, *Δp* < 10 kPa.

## CRediT authorship contribution statement

**Alireza Shantia:** Conceptualization, Methodology, Software, Validation, Formal analysis, Investigation, Data curation, Writing – review & editing. **Wolfgang Streicher:** Conceptualization, Methodology, Writing – review & editing, Funding acquisition.

## Declaration of Competing Interest

The authors declare that they have no known competing financial interests or personal relationships that could have appeared to influence the work reported in this paper. The authors declare the following financial interests/personal relationships which may be considered as potential competing interests:

## Data Availability

Experimental and analytical datasets for pressure drop and flow distribution in a Z-type flat-plate solar absorber (Original data) (Mendeley Data).

## Appendix Supplementary materials

Image, application 1
